# Accelerating the Gas–Solid Interactions for Conductometric Gas Sensors: Impacting Factors and Improvement Strategies

**DOI:** 10.3390/ma16083249

**Published:** 2023-04-20

**Authors:** Hongchao Zhao, Yanjie Wang, Yong Zhou

**Affiliations:** Key Laboratory of Optoelectronic Technology and System of Ministry of Education, College of Optoelectronic Engineering, Chongqing University, Chongqing 400044, China

**Keywords:** conductometric gas sensors, metal oxide, accelerating the reaction speeds, external activation, nanostructure construction, composites engineering

## Abstract

Metal oxide-based conductometric gas sensors (CGS) have showcased a vast application potential in the fields of environmental protection and medical diagnosis due to their unique advantages of high cost-effectiveness, expedient miniaturization, and noninvasive and convenient operation. Of multiple parameters to assess the sensor performance, the reaction speeds, including response and recovery times during the gas–solid interactions, are directly correlated to a timely recognition of the target molecule prior to scheduling the relevant processing solutions and an instant restoration aimed for subsequent repeated exposure tests. In this review, we first take metal oxide semiconductors (MOSs) as the case study and conclude the impact of the semiconducting type as well as the grain size and morphology of MOSs on the reaction speeds of related gas sensors. Second, various improvement strategies, primarily including external stimulus (heat and photons), morphological and structural regulation, element doping, and composite engineering, are successively introduced in detail. Finally, challenges and perspectives are proposed so as to provide the design references for future high-performance CGS featuring swift detection and regeneration.

## 1. Introduction

With the ever-increasing expansion of human actions, consequent chemicals, transportation, and manufacturing produce a great number of hazardous gases. For example, ammonia (NH_3_), CO, and acetone are released from fertilizer production, automobile exhaust, and nail polish, respectively, which severely threatens the ecological balance and human health. According to the Occupational Safety and Health Administration (OSHA), xylene, CO, formaldehyde, and other pollutants with a concentration of 200 ppm or higher are toxic to the respiratory tract. In the field of medical diagnosis, some volatile organic compounds (VOCs) from exhaled breath could serve as potential biomarkers of human diseases. For example, diabetics typically exhale higher concentrations of acetone than healthy individuals [1]. Among various gas detection technologies, conductometric gas sensors (CGS) mainly based on metal oxide semiconductors (MOSs) showcase brilliant superiorities of fast response, low cost, facile integration and miniaturization, and convenient operation over their electrochemical, optical, mass-sensitive, and photo-acoustic counterparts [2].

**Figure 1:**
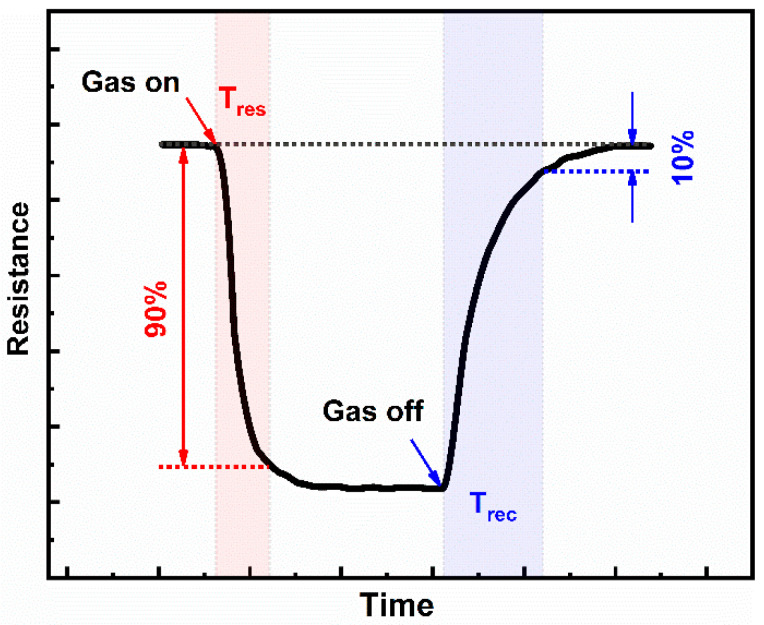
The response time and recovery time of a typical MOS gas sensor.

Of multiple operation parameters for a qualified CGS, the reaction speeds, including response time (usually termed as T_res_ or T_RS_(90)) and recovery time (usually termed as T_rec_ or T_RC_(10)), are very important, especially when applied within some urgent scenarios such as explosive and flammable environments. The quick response and recovery speeds during gas–solid interactions were critical for swift recognition of the target molecule so as to instantly enact management plans for the crisis and real-time monitoring of the dynamic environments to make prompt solution adjustments. As for their definitions, T_res_ and T_rec_ usually represent the time interval required to reach 90% of the maximum response during the adsorption process and 10% upon the desorption process, respectively. The shorter the response and recovery time, the faster the establishment of the adsorption/desorption balance. Figure 1 schematically shows the response and recovery times of a typical MOS gas sensor within one adsorption/desorption period [3]. Note that a majority of current MOS-based gas sensors suffer from slow response/recovery speeds, which severely hinders their further practical application [4,5,6]. Thus far, various strategies have been widely discussed, primarily in terms of response improvement. However, there have been no comprehensive reviews of influencing factors and improvement strategies with respect to the acceleration of response and recovery speeds of MOS gas sensors to date. Therefore, we focus on summarizing relevant reports here for the sake of paving the way for future research to overcome these obstacles. The discussion sequence is organized as follows. In Section 2, the factors impacting the response and recovery speeds of MOS gas sensors as well as related mechanisms, are categorized and discussed. Section 3 describes some potential strategies to improve the response and recovery features of MOS gas sensors. These strategies include external stimuli such as photo-activation and thermal excitation, subtle adjustment of nanomaterial grain size, porosity, hierarchical structure, element doping, composites engineering, etc. Section 4 offers a brief summary of current challenges and perspectives in this area.

## 2. Influencing Factors

### 2.1. Type of Gas-Sensitive Materials

Different MOSs possess a unique series of intrinsic characteristics, such as majority carrier concentration, defects, terminal groups, band gap, and electronic structure, which pose various influences on the gas–solid reactions. Therefore, a suitable design of the material system is quite critical for gas sensing. In this regard, N-type MOSs such as SnO_2_, ZnO, Fe_2_O_3_, etc., rather than P-type ones, are the first choice [7]. Generally speaking, the resistance change was more obvious upon gas adsorption for N-type MOSs compared to P-type ones [8] due to abundant inter-grain boundaries, which are conducive to trace gas detection and larger sensitivity. By contrast, the resistance modulation of P-type MOSs is essentially faint owing to the parallel conduction pathways at the interfaces of adjacent P-type nanoparticles [8,9,10]. Moreover, it has been proposed that the sensitivity of P-type MOS gas sensors is limited to the square root of N-type counterparts [10,11]. When detecting reducing gases with N-type MOSs, ambient oxygen molecules were first attached to the material surface and dissociated in the form of ionic oxygen species relying on the specific operation temperature, which consequently increased the baseline sensor resistance by reducing the electron density of the N-type MOS. After evacuating the reducing gases, the sensor resistance was restored to the initial value. At the same time, some P-type MOSs have been widely used in the field of gas sensing, mostly owing to their excellent catalytic capabilities, such as NiO, CuO, Co_3_O_4_, Cr_2_O_3,_ and so on [12,13,14,15]. Highly sensitive and selective P-type MOS gas sensors can be designed by doping/loading oxide or noble metal catalysts to facilitate the sensing reaction of specific gases. In addition, various gas sensors can be prepared using oxide p-n junctions, and reliable N-type MOS gas sensors can be designed by loading with P-type oxide additives [9]. When selecting the desired sensitive materials, an appropriate band gap should be preferentially considered (the desired bandgap is 2–4 eV)[16]. That is because, for the MOS with a large band gap, the electron hopping between its valence and conduction bands is difficult, thus resulting in a slight resistance change induced by gas adsorption. For the MOS with a small band gap, the resistance change is readily invisible due to the originally high carrier concentration and limited adsorption-induced concentration modulation. Single materials, regardless of N- or P-type MOS, always suffer from constrained sensitivity and severe cross-sensitivity and have to be combined with secondary materials (another metal oxide, noble metals, or layered nanomaterials) to avoid these issues, which uncertainly affects the reaction speeds at the same time.

In addition, according to reports in recent years, various MOS sensors have been utilized for the detection of defined gases. For example, ZnO, SnO_2_, and WO_3_-based sensors are often widely used for the detection of typical gases, including NO_2_, H_2_S, H_2_, NH_3_, and VOCs [17,18,19,20,21]. CuO-based sensors also show excellent sensing performance for H_2_S. Unlike most reducing gases, H_2_S often alters the surface composition of the host material, which is the so-called sulfur-poisoning effect. At the same time, H_2_S sensors based on CuO can convert CuO into metal-like CuS with higher conductivity [18]. For hydrogen sensing applications, hydrogen sensors with short response times of about 1 s and good recovery times can be improved using optimal Pd modifications [19]. For example, the SnO_2_ sensor loaded with 10mol% Pd developed by Li et al. [22] had a response/recovery time of only 4/10s toward 0.3% hydrogen at 200 °C. On the one hand, Pd nanoparticles benefited from the spillover effect and enhanced the dissociation of hydrogen molecules, leading to greater adsorption of hydrogen. On the other hand, the formation of the Schottky barrier between SnO_2_ and Pd and the reduction in the barrier height in the presence of hydrogen led to significant changes in the resistance of the gas sensor. For VOCs detection, In_2_O_3_, TiO_2,_ and α-Fe_2_O_3_ are also promising gas-sensitive materials with detection limits of a few parts per billion. Among different VOCs, ethanol and acetone detection has been widely used. Other important VOCs, such as benzene and acetylene, are rarely reported because they are difficult to exchange electrons, even under heating conditions [21].

### 2.2. Size Effect

#### 2.2.1. Thickness of Sensitive Layer

**Figure 2:**
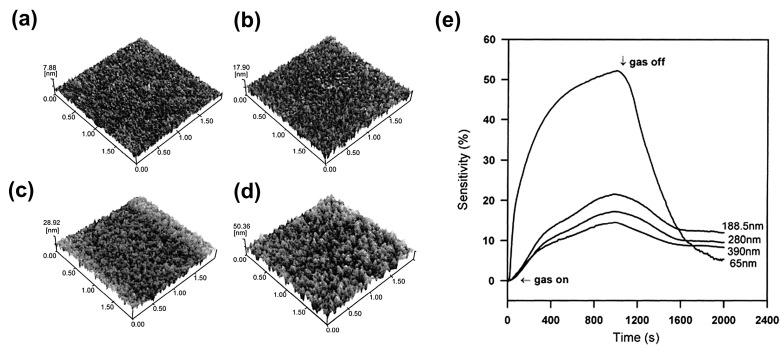
AFM micrographs (2 μm × 2 μm) of the as-deposited AZO films with various thicknesses: (**a**) 65 nm, (**b**) 188.5 nm, (**c**) 280 nm, and (**d**) 390 nm, (**e**) the effect of film thickness on the dynamic response toward 1000 ppm CO at 300 °C. Reprinted with permission from Ref. [27]. Copyright 2002, Elsevier.

The thickness of the sensitive layer poses a great influence on the sensor performance [23,24,25]. As the surface-controlled gas–solid reaction predominates the sensing behavior of CGS [26], the increase in film thickness adversely leads to harder gas diffusion and penetration into the lower layer due to the bulk hindrance effect, thus necessitating extra time to achieve the adsorption/desorption equilibrium [25]. For example, Al-doped ZnO (AZO) thin films with a thickness ranging from 65 to 390 nm were prepared by RF magnetron sputtering to detect 1000 ppm CO gas, as shown in Figure 2a–d [27]. When CO gas was switched off, the 65 nm thick films achieved the quickest response/recovery among all samples (Figure 2e). However, the film thickness is impossible to reduce without limitation, which is subject to the structural characteristics of the material itself and the specific film-deposition technology. Therefore, the film thickness should be optimized comprehensively, taking into account the response/recovery speeds, sensitivity, and preparation cost.

#### 2.2.2. Grain Size and Pore Size

**Figure 3:**
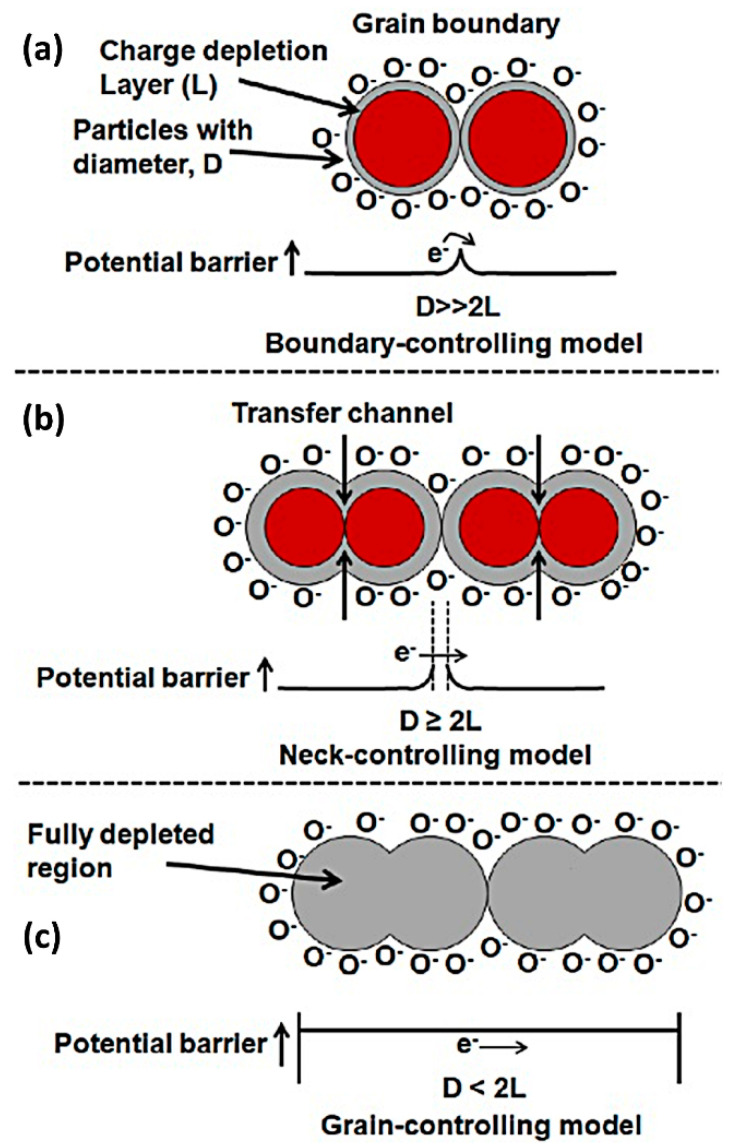
Schematic model of crystallite size effect on the sensitivity of MOS gas sensors: (**a**) D ≫ 2L, (**b**) D ≥ 2L, and (**c**) D < 2L. Reprinted with permission from Ref. [31]. Copyright 2022, Elsevier.

The grain size (D) is an important factor affecting the gas adsorption/desorption process and is closely related to the sensitivity and the response/recovery times. In particular, nanoscale grains benefit large specific surface area, abundant crystal faces, and thus sufficient gas adsorption. There is an exemplary relationship between grain size and the width of the space charge region (Debye length: L) [28,29,30], wherein three different cases were considered for the structure-property relationship. Typically, ambient oxygen adsorption of N-type MOSs produced a charge depletion layer near the surface, as displayed in Figure 3, when D ≫ 2L, the conductivity of the sensing material depended on the internal freely-moving charge carriers and was only influenced by the barriers between the grains. When D ≥ 2L, the boundary barriers, as well as the cross-sectional area of the narrow conduction channel between adjacent grains, determined the conductivity. When D < 2L, nearly every grain was fully depleted of electrons; since the conductivity was essentially controlled by the intergranular electron transport in this case, only a small amount of charge carriers during the surface reaction could cause a large variation in the conductivity of the whole structure. Meanwhile, small grain size brought about abundant interfaces containing multiple active sorption sites to anchor gas molecules and led to the swift establishment of dynamic adsorption/desorption balance, significantly accelerating the response/recovery speeds.

We can use the degree of depletion layer (*L*) to determine the electron conduction between adjacent grains of the material, as shown in Equation (1), where *ε, ε*_0_*, k, T, e,* and *n_b_* denote the dielectric constant, the permittivity in the vacuum, the Boltzmann constant, the temperature in Kelvin, the elementary charge, and the concentration of electrons in the material, respectively.

(1) $$L={\left(\frac{\varepsilon \varepsilon_{0}kT}{e^{2}n_{b}}\right)}^{1/2}$$

Smaller grain sizes are more favorable to improving the gas sensitivity. It has been shown in most cases that when the microcrystal size is downscaled below about 20 nm, the response is dramatically increased [32]. That is because the specific surface area of the sensitive material is remarkably enlarged when reducing the grain size at the scale of nanometers. Consequently, more active sites could be available to generate ionic oxygen species and chemical reactions, thus improving the gas-sensing performance.

In addition, pore size is also closely correlated with the response and recovery rates of gas sensors [33]. During the response and recovery process, the gas diffusion and penetration within the porous material are largely dependent on the pore size. The diffusion modes include surface diffusion, Knudsen diffusion, and molecule diffusion [34], and the corresponding diffusion constant increases with the increase in pore size. For example, the diffusion of target gas within the mesoporous material is dominated by Knudsen diffusion. The diffusion constant *D_k_* can be expressed as Equation (2), where *r*, *R*, *T,* and *M* represent the radius of the pore, the gas constant, the temperature of the diffused gas, and the molecular weight, respectively [34]. It can be concluded that the large diffusion constant of large pore size readily resulted in a quick adsorption/desorption balance. Varghese et al. [35] found that the gas sensors based on highly ordered nanoporous Al_2_O_3_ thin film with the largest pore size showed the shortest response/recovery times. Moreover, it is much easier for a gas molecule of a smaller kinetic diameter to go through the pores of a fixed size. Therefore, a trade-off between sensitivity and reaction speeds could be attained after reasonable modulation of the grain size and pore size.

(2) $$D_{k}=\frac{4r}{3}\sqrt{\frac{2RT}{\pi M}}$$

#### 2.2.3. Morphology and Structure

Since the gas-sensing response of MOSs mainly depends on the redox reaction on the surface, the surface morphology and nanostructure of the materials are very critical to determine the reaction kinetics of related gas sensors. The morphology difference lies in porosity, specific surface area, and reactive surface sites that affect gas adsorption, diffusion, reaction, and desorption. In general, sensing materials with hollow, porous, core-shell, or nanosheet-like morphology [36,37,38] harbor a large degree of porosity and high specific surface area, which is beneficial for facilitating gas transport within the materials and accelerating the gas–solid reactions [39,40]. In addition, low-dimensional nanomaterials could effectively suppress the scattering behavior of charge carriers and lengthen their mean free path as compared to bulk materials, also contributing to a fast response/recovery feature. These special structures are often prepared by hydrothermal and electrospinning techniques at low cost [41,42,43].

## 3. Improvement Strategies

Based on the above-influencing factors, the strategies to facilitate the response/recovery speeds are further discussed below.

### 3.1. External Excitation

In order to accelerate the reaction kinetics, additional energy inputs, primarily in the forms of heating and light irradiation, are adopted to stimulate molecular adsorption/desorption, simultaneously boosting the concentration change of free carriers within the materials [44,45]. The details about the temperature and light intensity and how response time was changed under the application of external stimulus are further discussed in Section 3.1.1 and Section 3.1.2.

#### 3.1.1. Thermal Excitation

**Figure 4:**
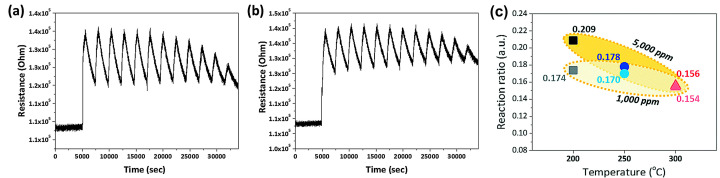
Performance of the CuO sensor: (**a**) The repeatability results for the CO gas-sensing response at 300 °C: run #1 and (**b**) run #2. (**c**) Reaction ratio for CO gas concentrations of 1000 and 5000 ppm. Reprinted with permission from Ref. [12]. Copyright 2019, The Royal Society of Chemistry.

Thermal excitation means that the operation temperature is raised to some extent when the sensor is working so that the interaction between gas molecules and sensitive materials can be strengthened and accelerated. In general, the response and recovery times of the as-tested sensors depend on the operation temperature because sufficient thermal energy is indispensable to overcome the activation energy of the redox reactions on the material surface and stimulate more free carriers within the MOS for stronger charge exchange [46]. As the concentration of majority carriers within MOSs is very small at room temperature, available reactive oxygen species are rare under this condition, severely limiting the gas-solid interactions. Moreover, the gas adsorption/desorption kinetics is quite slow due to the slow gas diffusion and penetration merely by dynamic gas flowing. As a result, MOS gas sensors routinely exhibit poor sensitivity and long response/recovery times in this case [2]. In particular, ZnO, SnO_2_, WO_3_, and In_2_O_3_-based gas sensors always operate over a temperature range of 200 to 400 °C for the sake of high sensitivity and swift reaction speeds [47,48,49,50]. For example, Tong et al. used a one-step oxidation method to prepare a TiO_2_ nanotube array thin film-based H_2_S gas sensor [3] and achieved response/recovery times as short as 22/6 s at 300 °C. Choi et al. [12] prepared 200 nm thick CuO film to detect 1000 to 5000 ppm CO at 200, 250, and 300 °C, respectively. The sensor exhibited the response and recovery times of 600 and 1800 s at 300 °C (Figure 4a–c) regardless of the CO concentration but at the expense of response intensity. It could be obtained that the response toward CO was inversely proportional to the operating temperature. This was mainly due to the low amount of pre-absorbed oxygen and the weak adsorption of CO gas on the CuO surface at high temperatures. Therefore, the response/recovery speeds cannot be markedly promoted by mere heating in some practical applications, despite ignoring other performance parameters.

#### 3.1.2. Light Activation

**Figure 5:**
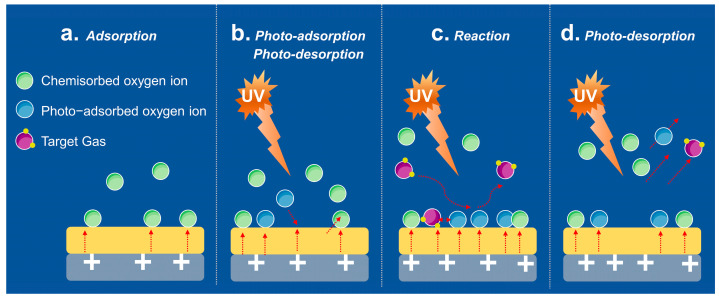
Photo-activated gas-sensing mechanism under UV irradiation.

Compared to thermal excitation, which has certain limitations, such as the complexity of sensor design and high energy consumption, photoexcitation is an economically viable solution. Due to the significant increase in the number of electron-hole pairs under light activation, photoexcitation is effective in facilitating the gas adsorption/desorption process and surface reactivity [45,51,52,53]. This strategy involves not only ultraviolet (UV) light [54,55,56,57,58,59,60,61,62] but also visible light [45,51,52,63,64,65,66,67]. In addition, light irradiation can significantly reduce energy consumption and avoid the safety concerns caused by high temperatures. A typical gas-sensing mechanism under ultraviolet irradiation is shown in Figure 5. In dark conditions, molecular oxygen from the ambient environment was attached to the surface of the sensitive material and created a high-resistance electron depletion layer near the material surface. When the incident light with higher energy than the bandgap of the material irradiated the material surface, abundant reactive electron-hole pairs were generated, thus leading to increased conductivity. At the grain boundaries, the excess photogenerated holes will react with the adsorbed oxygen ions to lower the barrier height. On exposure to the target gas, the gas molecules reacted with the active electrons (inherent and photogenerated electrons) and obviously altered the conductivity of the material [68].

**Figure 6:**
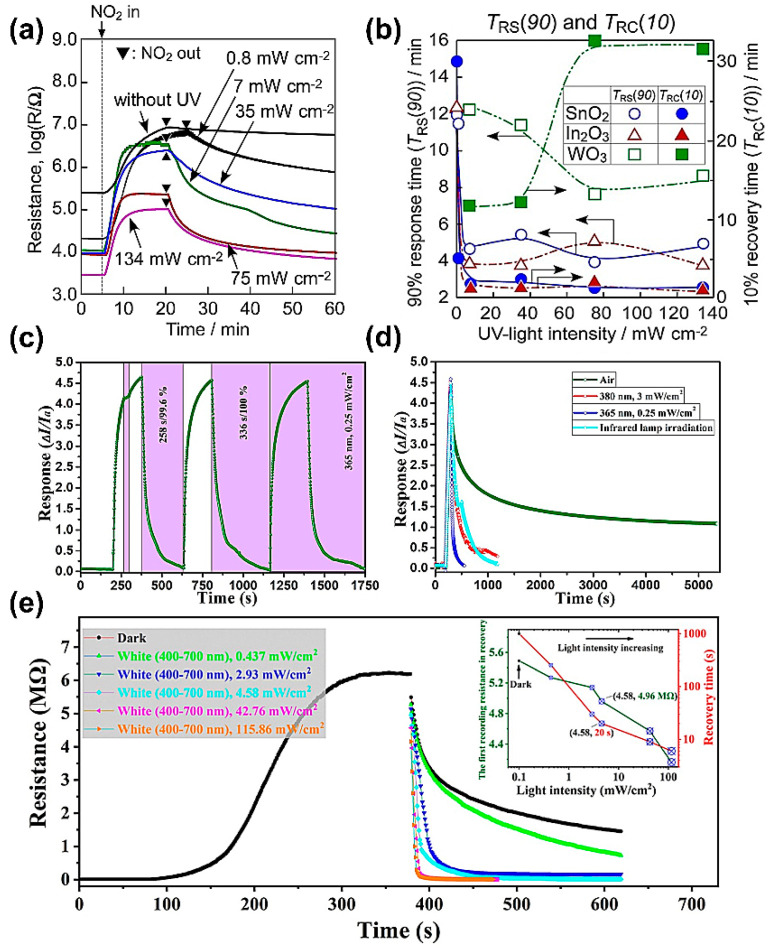
(**a**) Response transients of SnO_2_ sensor to 5 ppm NO_2_ at 30 °C in dry air under different UV-light irradiation intensities. (**b**) 90% response (T_RS_ (90), open symbols) and 10% recovery times (T_RC_ (10), filled symbols) of SnO_2_ sensor with UV-light intensity, together with those of In_2_O_3_ and WO_3_ sensors. Reprinted with permission from Ref. [69]. Copyright 2017, Elsevier. (**c**) Response and recovery curves of rGO-CeO_2_ sensor to 10 ppm NO_2_ with or without 365 nm UV-light irradiation and (**d**) comparison of different recovery methods. Reprinted with permission from Ref. [70]. Copyright 2018, Elsevier. (**e**) The response (communally) and recovery curves toward 5 ppm NO_2_ at room temperature of the In_2_O_3_ sensor in dark or under visible light irradiation with increasing intensity. The illustration displayed the variation trend of the recovery time and the first recording resistance in recovery with increasing light intensity. In the illustration, a larger blue symbol represents a stronger light. Reprinted with permission from Ref. [71]. Copyright 2021, Elsevier.

The UV-activated NO_2_-sensing properties of metal oxide (In_2_O_3_, SnO_2_, or WO_3_)-based gas sensors were investigated by Hyodo’s team [69]. It was found that the response/recovery behavior was strongly dependent on the light intensity. Figure 6a depicts the dynamic response of the SnO_2_ sensor toward 5 ppm NO_2_ as a function of light intensity within an isolated cycle, while Figure 6b shows the response and recovery times of all sensors as a function of UV light intensity. A very low response speed (about 12 min) with no recovery in the dark occurred. When increasing the light intensity to 7 mW cm^−2^, the recovery time was effectively reduced, possibly due to the promoted desorption of NO_2_ from the SnO_2_ surface. However, there was little correlation between the response time and light intensity over the range of 7–134 mW cm^−2^. Therefore, the irradiation intensity of UV light played an important role in improving the response and recovery speeds.

Moreover, it was found that the full recovery of the sensor was not achieved even with stronger UV irradiation. In most cases, gas sensors could be completely reversible under ultraviolet light activation at room temperature [70,72,73,74]. For example, Hu et al. [70] reported that the recovery time of a NO_2_ sensor based on reduced graphene oxide (rGO)-CeO_2_ hybrids could be greatly reduced from 10 min under the dark case to 258 s under ultraviolet light of 0.25 mW cm^−2^ (Figure 6c,d). That is because UV light favors gas desorption by breaking the high-energy bonds between the sensitive material and target gas molecules. Zaporotskova et al. [75] designed a new sensor to detect gases and organic vapors at room temperature with a detection limit as low as 44 ppb for NO_2_ and 262 ppb for nitrotoluene, respectively. However, due to the existence of high-energy bonds between carbon nanotubes and some gas species, the recovery time was as long as 10 h under the dark case. By applying UV radiation, the recovery time was obviously reduced to 10 min.

Although the energy of UV light is greater than that of visible light, using excessively energetic photons that exceed the threshold of the band gap of the sensitive material does not necessarily bring about a better response. This is a result of inelastic scattering, where a greater proportion of conduction electrons are lost as the energy increases [76]. Therefore, photons whose energy is close to the material’s band gap could lead to optimal gas-sensing properties. That is also why semiconductor layers can be activated by visible light (VL) [68]. For example, Zhang et al. [71] successfully solved the issue of the slow NO_2_-recovery process of the In_2_O_3_ sensor at room temperature by introducing visible light irradiation (400–700 nm). As shown in Figure 6e, in addition to the response time of 200 s toward 5 ppm NO_2_, the sensor’s recovery time was nearly a quarter (253 s) shorter than the recovery time (1000 s) under dark conditions, even with the introduction of weaker light intensity (0.437 mW/cm^2^). Striking recovery performance was also observed when the light intensity was enhanced to 42.76 mW/cm^2^ or higher values (115.86 mW/cm^2^), respectively, yielding recovery times of 9 and 6 s. The photocurrent generation under visible light irradiation promoted the rapid desorption of NO_2_ molecules. However, the sensor resistance was unstable after the removal of visible light with excessively strong light intensities (42.76 and 115.86 mW/cm^2^). In contrast, when the sensor was exposed to visible light at 2.93 and 4.58 mW/cm^2^, the sensor resistance could maintain dynamic stability even when the light was turned off, indicating that it was fully recovered with the help of proper illumination intensity and the recovery time varied with the light intensity (Figure 6e). Eventually, the optimal light intensity of 4.58 mW/cm^2^ was determined by comparing the recovery rate of the sensor since the start of the recovery process (100% for 4.58 mW/cm^2^ vs. 97.43% for 2.93 mW/cm^2^).

### 3.2. Nanostructure Design

#### 3.2.1. Modulation of Size and Thickness

**Figure 7:**
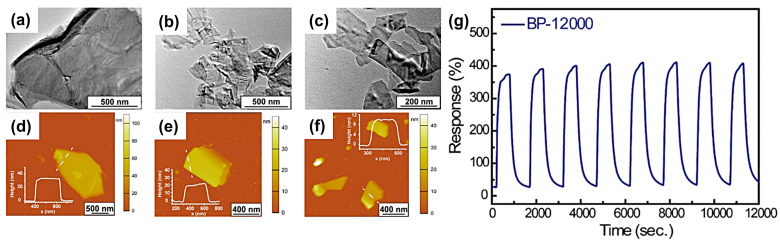
Characterization using (**a**–**c**) TEM images and (**d**–**f**) AFM graphs of BP-4000 (**a**,**d**), BP-7000 (**b**,**e**), and BP-12,000 (**c**,**f**) samples. (**g**) dynamic response of BP-12,000 under 1000 ppb NO_2_ exposure showing complete recovery over eight cycles. Reprinted with permission from Ref. [78]. Copyright 2020, American Chemical Society.

According to the analyses in the previous section, the response-recovery features of MOS gas sensors are highly dependent on the grain size of the MOS nanoparticles and the thickness of the related sensing film. In a previous report, Wang et al. [77] prepared 5% W-doped SnO_2_ nanoparticles to detect 10 ppm H_2_S and showed a response and recovery time of 17 and 7 s. It was found that the grain size was reduced from 15 nm of undoped SnO_2_ to 6 nm of 5% W-doped SnO_2_ while the electrical conductivity was increased. Moreover, the controlled thickness of the sensitive layer was beneficial for the sensing recovery. Chen et al. [78] prepared black phosphorus (BP) nanosheets of various sizes by density-gradient centrifugation. The time of each centrifugation was fixed at 10 min. After the first centrifugation at 1000 rpm to remove the bulk BP crystals, the remaining supernatant was centrifuged a second time at 4000 rpm, which was labeled BP-4000. Similarly, the subsequent products centrifuged at 7000 and 12,000 rpm were specified as BP-7000 and BP-12,000, respectively. The lateral sizes of BP-4000, BP-7000, and BP-12,000 nanosheets are ~1 μm, ~600 nm, and ~300 nm (TEM images in Figure 7a–c), while the real sizes (thickness) were 1.5 μm × 1.2 μm (35 nm), 0.4 μm × 0.6 μm (21 nm) and 0.3 μm × 0.2 μm (12 nm) (AFM images in Figure 7d–f), respectively. Among them, the BP-12,000 sample with the smallest areal size and thickness could fully recover after exposure to 100 ppb NO_2_ at room temperature (Figure 7g).

In addition, Zhang et al. [79] found that the thinner SnO_2_ nanosheets exhibited better response and recovery characteristics than their thicker counterparts. It was claimed that thinner nanostructures possessed more surface defects (especially oxygen vacancies) that allowed more oxygen adsorption and led to a wider electron depletion layer and then better sensor response [80]. The rapid response/recovery could be explained by the comparable thickness to the Debye length (L). For SnO_2_ in air, L_D_ value was reported within the range from about 3 nm [81] to 7 nm [82,83]. For a thickness smaller than 2L, the charge depletion region would extend throughout the whole nanosheets, resulting in a swift conductivity stabilization when interacting with the target gas.

#### 3.2.2. Construction of Porous and Hierarchical Structures

**Figure 8:**
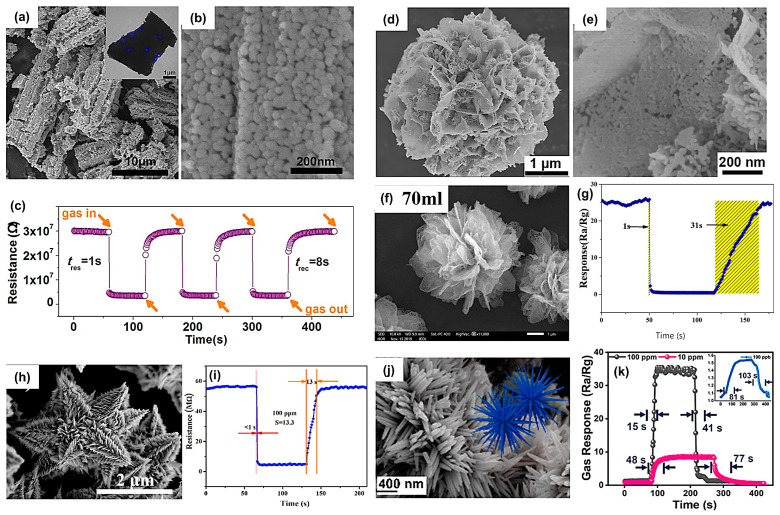
The typical FESEM images (**a**,**b**) In_2_O_3_ hierarchical architectures. The inset is the TEM image of In_2_O_3_ hierarchical architectures. (**c**) Transient responses of In_2_O_3_ hierarchical architectures to 100 ppm formaldehyde at 260 °C. Reprinted with permission from Ref. [86]. Copyright 2018, Elsevier. SEM images of (**d**,**e**) 0.3% Pt–SnO_2_. Reprinted with permission from Ref. [87]. Copyright 2020, Elsevier. (**f**) SEM of porous flower-like α-Fe_2_O_3_ with 70 mL ethylene glycol. (**g**) Response and recovery curve of α-Fe_2_O_3_ with 70 mL ethylene glycol to 100 ppm acetone at operating temperature 210 °C. Reprinted with permission from Ref. [40]. Copyright 2020, Springer. (**h**) FESEM images of the cedar-like SnO_2_ micro-nanostructure. (**i**) The transient response of cedar-like SnO_2_ sensors exhibits to 100 ppm formaldehyde at 200 °C. Reprinted with permission from Ref. [88]. Copyright 2017, Elsevier. Typical FESEM image of (**j**) Zn-doped layered SnO_2_ nanocones sample. (insets) Schematic illustration of the structure unit of the sample. (**k**) The transient response and recovery times of the Zn-doped layered SnO_2_ sensor toward three different concentrations of TEA (100 ppb, 10 ppm, and 100 ppm) at 70 °C, 57% RH. Reprinted with permission from Ref. [89]. Copyright 2020, American Chemical Society.

Porous and hierarchical nanostructures enable a rapid diffusion and transport of gas molecules within sensitive materials and have been attracting ever-increasing attention in the field of gas sensors [84,85]. These structures not only enhance the interaction with the target analyte through enough active sorption sites but also facilitate the sensing response and recovery by virtue of their high porosity. For example, Wang et al. [86] prepared a porous In_2_O_3_ structure with abundant oxygen vacancies and a large specific surface area (Figure 8a,b). The synergistic effect of oxygen vacancies and grain boundaries contributed to an improved gas sensitivity toward formaldehyde (HCHO), excellent linearity within the concentration range of 5–100 ppm at 260 °C and fast response/recovery toward 100 ppm HCHO (1 s/8 s) (Figure 8c).

Likewise, the flower-like structure was favorable for gas sensing [87], as shown in Figure 8d,e. These morphologies typically exhibited a high specific surface area of 18.2 to 156 m^2^g^−1^ and pore sizes of 7.8 to 31.2 nm. The combination of internal and external voids benefited a large specific surface area and faster response time (recovery time) ranging from 8 to 26 s (1 to 16 s) at 120 to 300 °C [31]. In addition, Zhang et al. [40] prepared a series of α-Fe_2_O_3_ samples and tuned the morphology by changing the amount of added ethylene glycol during the synthesis process. When the added amount was increased from 30 mL to 110 mL, the product assembled by the two-dimensional nanochip was converted into a three-dimensional porous α-Fe_2_O_3_ micro flower. In particular, when the dosage was 70 mL, the product possessed the most uniform structure, the largest specific surface area (63.69 m^2^/g), the most oxygen vacancies, and the shortest response and recovery times toward 100 ppm acetone (1 s/31 s) (Figure 8f,g). The quick reaction kinetics were mainly attributed to the decrease in grain size, the enlarged specific surface area, and sufficient sorption sites to accommodate a large number of gas molecules within a short period.

As presented in another work, a novel three-dimensional cedar-like SnO_2_ of a stem section and a leaf-like structure located around the stem was prepared, as shown in Figure 8h [88]. This open structure allowed free gas diffusion and exhibited high sensitivity and fast response/recovery times toward HCHO detection. Remarkably, the response/recovery times for 100 ppm HCHO were <1 s/13 s (Figure 8i).

Similarly, the synthesis of Zn-doped layered SnO_2_ decahedrons, nanorods, and nanocones (Figure 8j) was conducted by Wang et al. [89]. The different nanostructures were realized by varying the Zn content. The rapid response and recovery toward triethylamine (TEA) at 70 °C (Figure 8k) were primarily attributed to the high-energy crystal plane (101) developed at lower Zn content, which promoted ion adsorption of oxygen and optimized the electronic structure of host SnO_2_ material.

Now that the discussion of nanostructure design had been completed, the characteristics of different photoactive materials (In_2_O_3_, SnO_2_, or WO_3_) mentioned in Section 3.1.2 were systematically compared in Table 1, including their typical sensing characteristics toward 5 ppm NO_2_ at 30 °C in dry air under an optimal UV-light irradiation (365 nm) of 7 mW cm^−2^ as well as their crystal structure and specific surface area.

According to Hyodo’s team [69], the effects of UV irradiation on the NO_2_ sensing characteristics of SnO_2_, In_2_O_3,_ and WO_3_ sensors were similar, but there were some essential behavior differences among them. The resistance of the WO_3_ sensor was the highest, and that of the In_2_O_3_ sensor was the lowest regardless of UV irradiation. The resistance of all three sensors decreases as the intensity of ultraviolet light increases. In addition, ultraviolet light can effectively reduce their response and recovery time. The In_2_O_3_ sensor had the highest NO_2_ response among all sensors without UV irradiation. However, even weak UV irradiation greatly reduced the NO_2_ response of the In_2_O_3_ sensor, which decreased with increasing UV intensity. As a result, the In_2_O_3_ sensor was much less responsive to NO_2_ under UV light than to SnO_2_ sensors. The NO_2_ response of the WO_3_ sensor could not be confirmed due to the limitation of the experimental conditions of the researchers because of the high resistance without UV irradiation. In the absence of UV irradiation, the NO_2_ response of the WO_3_ sensor was greater than 900, which was greater than the SnO_2_ sensor. UV irradiation significantly reduced the NO_2_ response of WO_3_ and was the smallest among all sensors. In addition, WO_3_ sensors responded and recovered much more slowly than SnO_2_ and In_2_O_3_ sensors, even under UV light. Combined with our discussion on morphology, the specific surface area of WO_3_ less than SnO_2_ and In_2_O_3_ may also be the reason for the low response. These results indicated that although UV light played a role in response/recovery speed, In_2_O_3,_ and WO_3_ sensors were far less suitable for sensitive detection of NO_2_ under UV light than SnO_2_ sensors from a response perspective. The researchers also speculated that they might be able to detect NO_2_ and other gases more sensitively under visible light since In_2_O_3_ and WO_3_ generally have lower band gaps (~2.7–2.8 eV) than SnO_2_ (~3.6 eV) [31,90]. This was also consistent with previous studies on NO_2_ sensors based on In_2_O_3_ and WO_3_, which exhibited better sensitivity and faster response/recovery under irradiation of 439 nm and 430nm, respectively, unlike sensors based on SnO_2_ under irradiation of 365 nm [91,92].

### 3.3. Element Doping

Doping can effectively modify the electronic structure and surface reactivity of the host MOS material. First, doping from the donors or acceptors can produce extra free electrons or holes that can react with ambient oxygen or the target gas molecules. Second, alien atom incorporation could introduce a new energy level and alter the transportation environment of the charge carriers. Meanwhile, reactive dopants readily trigger surface-catalyzed reactions and enhance the response and recovery [6,49,93,94,95].

**Figure 9:**
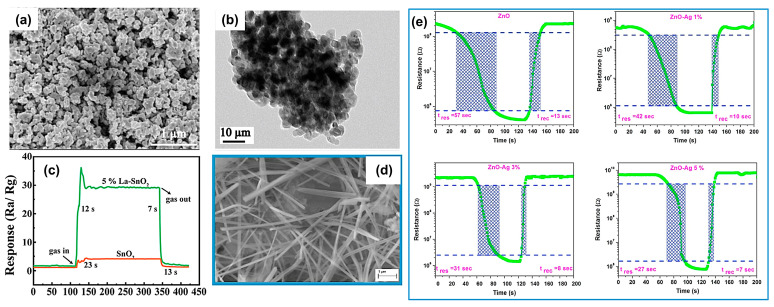
SEM images, TEM images of 5 wt% La-doped SnO_2_ nanocomposite (**a**,**b**). (**c**) Dynamic response-recovery curves of pure and 5 wt% La-doped SnO_2_ against 75 ppm methanol at 220 °C. Reprinted with permission from Ref. [98]. Copyright 2020, Springer. (**d**) SEM images of ZnO:Ag thin films with 5 wt% Ag doping. (**e**) Response time and recovery time of ZnO:Ag (0–5%) thin film deposited on a glass substrate with exposure and removal of NH_3_ gas of 25 ppm concentration. Reprinted with permission from Ref. [99]. Copyright 2020, Elsevier.

For example, doping of the lanthanide elements is capable of raising the gas sensitivity and reaction speeds of SnO_2_ for VOC detection because these elements actively participate in complex surface reactions. There were similar cases of Eu-doped SnO_2_ nanofibers [96] and nanorods [97]. As displayed in Figure 9a,b, the 5 wt% La-doped SnO_2_ reported by Chen et al. [98] showed quick response/recovery speeds toward 75 ppm methanol (12 s/7 s) than those (23 s/13 s) of undoped SnO_2_ (Figure 9c). These improvements were mainly ascribed to the decrease in grain size from 9.2 to 5.5 nm after La doping, which enabled a larger surface area with more active sites for oxygen and methanol adsorption.

In addition, noble metal doping is also a common strategy to improve the response and recovery characteristics of gas sensors [93,99]. Li’s team [93] prepared Pd_x_W_18_O_49_ nanowires (NWs) via a simple hydrothermal method. It was found that the concentration of oxygen vacancy first increased with increasing Pd content and reached the maximum percentage (52.5%) at a Pd content of 7.18 wt% (Pd_7.18%_W_18_O_49_ NWs). The Pd content was determined by ICP–AES (Inductively Coupled Plasma–Atomic Emission Spectroscopy) result. Further, an increase in Pd content brought about decreased oxygen vacancies. As is well known, oxygen vacancies can be used as good adsorption sites or active catalytic sites to promote the gas-solid interaction [100]. Therefore, the Pd_7.18%_W_18_O_49_ NWs showed the largest response and the fastest response/recovery speeds (5 and 10 s) for acetone detection at 175 °C. Kamble et al. [15] investigated the effect of doping with trace Pt metal on the gas-sensing properties of Cr_2_O_3_ films. The results show that Pt doping reduced the adsorption and desorption energy, which not only enhanced the sensor response but shortened the response/recovery times. Devi et al. [99] prepared NH_3_ vapor sensors based on ZnO thin films doped with different Ag contents (Figure 9d). The response and recovery times of the 5 wt% Ag-doped ZnO sensor to 25 ppm NH_3_ gas at room temperature were 27 and 7 s (Figure 9e).

Nonmetal element doping, such as N, O, Si, etc., also plays analogous roles in gas sensing. Our group found that the doped N atoms could serve as good electron donors capable of activating the electron-transfer reactions and increasing the sorption sites [101,102,103]. In another report [104], hierarchical oxygen-doped MoSe_2_ nanosheets were obtained by mild calcination at different temperatures for 0.5 h. Compared with the pristine MoSe_2_, the resulting sample calcined at 200 °C (MoSe_2_-200) showed a very similar response time (~32 s) and about a tenth of a reduction in recovery time (from 253 s to 25 s) toward 100 ppm TEA at room temperature. It was claimed that the structural change in the sensitive material after calcination and the difference in electronegativity between the non-metallic elements and the TEA molecules ensured the rapid recovery of MoSe_2_-200 nanosheets. Specifically, MoSe_2_-200, after calcination in air, was generally inert and did not react easily with O_2_. With the incorporation of oxygen that was inclined to efficiently bind the Mo atoms in MoSe_2_-200, the affinity of MoSe_2_-200 to TEA was correspondingly reduced due to the greater electronegativity of oxygen relative to that of nitrogen in TEA, which explained why adsorbed TEA molecules were easily desorbed from MoSe_2_-200 by fresh air.

### 3.4. Composites Engineering

#### 3.4.1. Heterojunction

In addition to element doping, constructing heterojunction nanostructures including n-n, n-p, or Schottky-type [12,54,55,105,106,107] between two different materials is a fascinating way of regulating the band structure and energy levels and then improving the sensor response and recovery.

**Figure 10:**
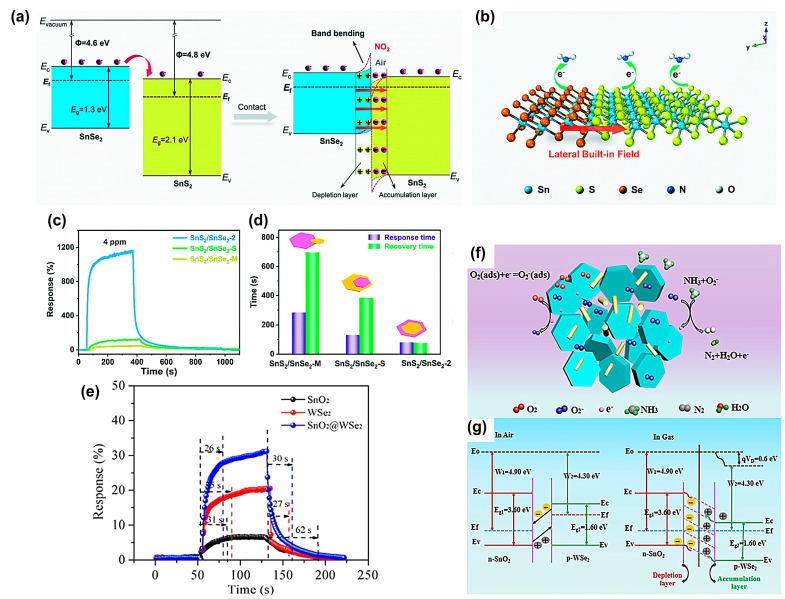
Schematic illustrations of (**a**) energy band structure and (**b**) NO_2_ adsorption for the 2D/2D SnS_2_/SnSe_2_-2 heterostructure. (**c**) Response and (**d**) response and recovery times of SnS_2_/SnSe_2_–M, SnS_2_/SnSe_2_–S, and SnS_2_/SnSe_2_-2 heterostructures toward 4 ppm NO_2_ at room temperature. Reprinted with permission from Ref. [108]. Copyright 2022, The Royal Society of Chemistry. (**e**) Response/recovery time of SnO_2_/WSe_2_, WSe_2,_ and SnO_2_ sensors upon exposure to 1 ppm NH_3_. (**f**) Energy-band structure of the SnO_2_/WSe_2_ sensor in air and NH_3_ gas (Ec, Eg, Ev, and Ef are the bottom of the conduction band, band gap, top of the valence band, and the fermi-energy level). (**g**) Illustration of NH_3_ sensing mechanism for SnO_2_/WSe_2_ nanocomposite. Reprinted with permission from Ref. [111]. Copyright 2022, Elsevier.

Wu et al. [108] synthesized in-plane SnS_2_/SnSe_2_ heterostructures with an atomic interface using a topologically oriented anion exchange method, in which the content and spatial arrangement of in situ grown SnSe_2_ could be controlled by adjusting Sn precursors. Compared with the common SnS_2_/SnSe_2_ heterostructures prepared by mechanical agitation and solvothermal deposition, the optimal SnS_2_/SnSe_2_ planar heterostructures achieved 3.5/8.9 times faster response and 1.6/4.9 times faster recovery time toward 4 ppm NO_2_ at room temperature, respectively (Figure 10c,d). Within the in-plane heterostructures, a partial subdivision of SnS_2_ template nanosheets was determined by transversely grown SnSe_2_ layers. Therefore, the transverse orientation of the in-plane heterojunction exposed highly active interfaces to the outside and then promoted the NO_2_ adsorption and surface charge exchange. More importantly, due to the intimate contact between SnSe_2_ and SnS_2_ crystals, a built-in field was formed (Figure 10b), which could significantly improve the charge transfer efficiency on the two-dimensional plane [109,110]. When NO_2_ molecules adsorbed and captured electrons from the conduction band of the material, the electron concentration of the sensitive material dropped sharply, disrupting the charge balance within the SnS_2_/SnSe_2_-2 planar heterojunctions (Figure 10a). More electrons diffused from SnSe_2_ into SnS_2_, quickly replenishing the lack of electrons in SnS_2_ under the strong built-in field, thus achieving a quick response/recovery.

A room-temperature NH_3_ sensor based on SnO_2_/WSe_2_ nanostructure synthesized by a hydrothermal method was fabricated [111]. The response/recovery speeds of SnO_2_/WSe_2_ composites toward 1ppm NH_3_ were faster than those of pure SnO_2_ or WSe_2_ counterparts (Figure 10e). This improvement was mainly attributed to the special structure of p-n heterojunctions between P-type WSe_2_ hexagonal nanosheets and N-type SnO_2_ nanorods. Their different work functions led to the electron transfer within the heterojunctions, which significantly improved the sensor performance (Figure 10f,g). In addition, WSe_2_ nanosheets also possessed a large specific surface area and provided a large number of adsorption sites, also effectively promoting NH_3_ adsorption and desorption [112,113].

**Figure 11:**
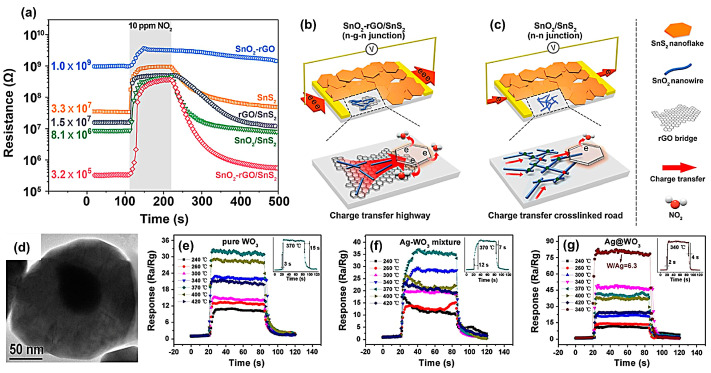
(**a**) Real-time resistance curves of SnO_2_-rGO, SnS_2_, SnO_2_/SnS_2_, and SnO_2_-rGO/SnS_2_ sensors under 10 ppm NO_2_ at 120 °C for comparison. Schematic illustration of charge transfer difference between (**b**) SnO_2_-rGO/SnS_2_ sensor with novel n-g-n heterojunctions and (**c**) SnO_2_/SnS_2_ sensor with traditional n-n junctions. Reprinted with permission from Ref. [116]. Copyright 2021, Elsevier. (**d**) TEM image of Ag@WO_3_ core-shell nanostructures with the core and the shell clearly resolved. Transient response at different temperatures for sensors using 100 ppm alcohol vapor exposure. The upper-right inset in each Figure shows corresponding response and recovery curves for the senor at its optimum working temperature. (**e**) Pure WO_3_, (**f**) Ag–WO_3_ mixture, and (**g**) Ag@WO_3_. Reprinted with permission from Ref. [107]. Copyright 2015, Elsevier.

Conventional tandem heterostructures probably possessed a lot of interfacial defects that probably hindered the charge transfer at these interfaces, thus resulting in a suppressed response [114,115]. Zheng et al. [116] proposed a novel layered assembly of SnO_2_-rGO/SnS_2_ heterostructures that exhibited a shorter response and recovery time (42 and 111 s) toward 10 ppm NO_2_ than the counterparts of a single component or binary composites, thus achieving an ultra-sensitive NO_2_ detection (Figure 11a). Herein rGO acted as a transfer highway connecting N-type SnO_2_ nanowire and N-type SnS_2_ nanowire, resulting in a novel ternary n-g-n junction that enabled the efficient transfer of electrons from SnO_2_ to SnS_2_ (Figure 11b). The transient photocurrent response, compared to n-n junctions (Figure 11c), indicated that the n-g-n junction using the bridged rGO optimized the carrier transport between SnO_2_ and SnS_2_. Furthermore, the large specific surface area and abundant edge sites of rGO enabled NO_2_ molecules to adsorb and diffuse around SnS_2_ material effectively, promoting electron transport from SnS_2_ to NO_2_.

Schottky-type heterojunction can be formed between noble metals and MOS. This heterogeneous interface also played a multifunctional role in the response and recovery rates of gas sensors. Xu et al. [107] prepared pure WO_3_, Ag-WO_3_ mixture, and Ag@WO_3_ core-shell nanostructures (Figure 11d) by a hydrothermal method and tested their sensing properties toward 100 ppm ethanol vapor. The response/recovery times were found to be significantly shortened, from 3/15 s for pure WO_3_ (Figure 11e), 12/7 s for Ag-WO_3_ mixtures (Figure 11f), to 2/4 s for Ag@WO_3_ core-shell nanostructures (Figure 11g). In addition, their optimal operating temperature was also reduced from 370 to 340 °C. The improved sensing performance was attributed to the formation of Schottky junctions at the Ag/WO_3_ interfaces, confirmed by a significant shift of the XPS Ag 3d peak in the Ag@WO_3_ sample compared to the pure WO_3_ one. Within the physical mixture of Ag and WO_3,_ which were separately prepared, however, the spacing between Ag nanoparticles and WO_3_ particles was too large to form Schottky-type junctions effectively and thus deteriorated the sensor performance.

#### 3.4.2. Quantum Dot Modification

**Figure 12:**
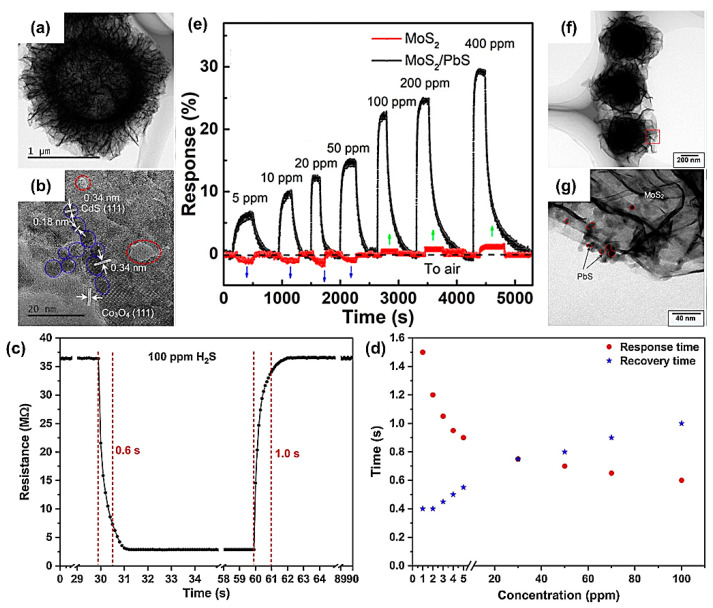
(**a**,**b**) TEM and HRTEM images of CdS QD/Co_3_O_4_ synthesized by in situ growth method at 25 °C. Performance of the CdS QD/Co_3_O_4_ sensor: (**c**) The response curve of the sensor to 100 ppm H_2_S at 25 °C, (**d**) Response and recovery times of the sensor to H_2_S at concentrations ranging from 1 to 100 ppm. Reprinted with permission from Ref. [36]. Copyright 2019, Elsevier. (**e**) Dynamic response–recovery curves of MoS_2_ and MoS_2_/PbS gas sensors without a heating device at 5–400 ppm NO_2_ concentrations. (**f**,**g**) TEM images of MoS_2_/PbS composites. Reprinted with permission from Ref. [124]. Copyright 2019, American Chemical Society.

Quantum dots (QDs) are considered zero-dimensional semiconductor materials whose size at three dimensions does not exceed twice the exciton Bohr radius of the corresponding semiconductor material and are noteworthy because of their size advantages. However, due to the poor permeability of QDs aggregates, QDs-based gas sensors always show relatively long response/recovery times [117,118,119,120,121,122]. For example, Liu’s team [123] realized an extremely high response (4218) to 50 ppm H_2_S at 135 °C based on PbS colloidal QDs, but with a relatively long response/recovery rate (23/171 s). To this end, combining QDs with secondary nanomaterial to form porous and layered structures can overcome the dilemmas. For example, Dun et al. [36] produced CdS QDs using hollow Co_3_O_4_ microspheres assembled from ultra-thin porous nanosheets as the support. As shown in Figure 12a,b, CdS QDs (about 4.5 nm) circled in blue were distributed on porous Co_3_O_4_ nanosheets circled in red. The prepared CdS QDs/Co_3_O_4_ sensor showed a super-fast response/recovery rate (0.6/1.0 s) toward 100 ppm H_2_S at 25 °C (Figure 12c), which was attributed to the high activity of QDs and hollow nanostructures. Figure 12d shows the response and recovery times as a function of H_2_S concentration from 1 to 100 ppm. The response time became shorter with concentration, as opposite to the recovery time. This also proved that the gas sensor achieved a quicker saturation at higher concentrations but at the cost of harder desorption.

Xin et al. [124] combined hydrothermal and chemical precipitation methods to synthesize PbS QDs-modified MoS_2_ composites (MoS_2_/PbS) (Figure 12f,g). The MoS_2_ sensor showed low responses for NO_2_ at all concentrations (5–400 ppm) and exhibited different response behaviors (negative response at 5–50 ppm and positive response at 100–400 ppm, shown as the blue and green arrows in Figure 12e, respectively), as well as incomplete recovery. By contrast, the MoS_2_/PbS composite devices exhibited a higher positive response to NO_2_ and complete recovery with almost no drift (recovery ratio was over 99%) (Figure 12e). This resulted from the large surface area of MoS_2_ and the close interaction between PbS QDs and NO_2_ molecules.

#### 3.4.3. Charge Transfer Improvement

Single MOSs usually exhibit the disadvantages of poor conductivity and slow recovery at low temperatures [125,126]. To overcome these difficulties, two-dimensional nanomaterials such as graphene (GO), rGO, and BP have been widely used to modify MOSs by virtue of their large specific surface area, excellent room-temperature conductivity, and customizable photoelectric properties [127,128,129,130,131,132]. For example, Peng and co-workers [129] prepared a high-performance room-temperature H_2_S gas sensor using rGO-modified WO_3_ nanocubes. The results unveiled that WO_3_/rGO composites attained the best performance when the content of rGO was 5 wt%, and the recovery time toward 0.5 ppm H_2_S was as fast as 180 s. The improved recovery could be attributed to the following reasons. On the one hand, the charge transfer efficiency was significantly raised due to the C-O-W chemical bonds that acted as the charge transfer bridge between WO_3_ and rGO interfaces. On the other hand, rGO nanosheets provided a large surface area to accommodate WO_3_ nanocubes and enhance the gas adsorption and diffusion, improved the efficiency of electron transfer, and speeded up the recovery rate of the sensor due to its excellent conductivity. Likewise, rGO nanosheets loaded with SnO_2_ nanoparticles [133] and Fe_2_O_3_ nanoparticles [134] showcased the same improvements.

#### 3.4.4. Ternary Composite

The combination of low-dimensional materials (such as carbon nanotubes or graphene derivatives), metal oxides, and noble metals for ternary composites has been verified to be particularly beneficial for enhancing gas-sensing properties [135,136,137].

The electrical and physical properties of individual materials, heterojunctions between different materials, and the synergy effect can all pose a positive impact on the response and recovery speeds of relevant gas sensors. For example, Ghosal et al. [138] prepared ternary Pd/rGO/MnO_2_ heterojunctions to investigate the response to methanol vapor. The results showed that the response time (11 s) and recovery time (16 s) toward 100 ppm methanol vapor were shorter than those of the binary (Pd/MnO_2_:36s, 43s; rGO/MnO_2_:21 s, 27 s) and pure MnO_2_ counterparts (59 s, 67 s). With regard to this improvement, Pd as a catalyst contributed to the easy dissociation of the target molecules and enabled an improved performance at low temperatures. Second, rGO ensured fast response/recovery kinetics due to its high carrier mobility. Moreover, three-dimensional MnO_2_ nanoflowers (NFs) provided a high specific surface area and a large gas adsorption capability.

These are the main approaches to improving sensing speed that we have discussed. We summarized the previous efforts to accelerate the response/recovery of gas sensors in Table 2, including some that may not be specifically mentioned in the text.

### 3.5. Other Strategies

Apart from the above methods, there are other strategies available to improve the reaction kinetics.

#### 3.5.1. Humidity

Generally speaking, humidity involvement during the gas-sensing process readily induces the reduction in the sensor response by occupying available sorption sites that should be originally interacted with target gas molecules [174].

**Figure 13:**
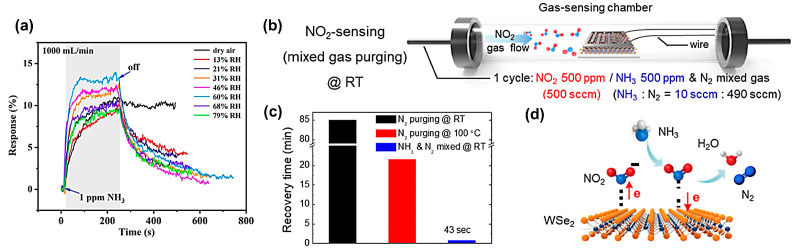
Performance of the N-MXene sensor: (**a**) humidity effect on sensor response toward 1 ppm of NH_3_. Reprinted with permission from Ref. [175]. Copyright 2021, American Chemical Society. (**b**) Schematic image of the gas-sensing chamber for enhanced WSe_2_ recovery. Gas delivery comprised NO_2_ at 500 ppm exposure (500 sccm) and mixed gas (NH_3_ and N_2_) purging (500 sccm). (**c**) Recovery time comparison of WSe_2_ sensor toward 500 ppm NO_2_ at different conditions: N_2_ purging @ RT, N_2_ purging @ 100 °C and NH_3_ and N_2_ mixed @RT (**d**) Schematic image of WSe_2_ sensor recovery for NO_2_ absorption and desorption using NH_3_ and N_2_ mixed purging gas. Reprinted with permission from Ref. [177]. Copyright 2018, American Chemical Society.

However, in some cases, humidity was a positive sensitization factor to accelerate the response/recovery speeds. Zhou et al. [175] studied the preparation of MXene Ti_3_C_2_T_x_-derived nitrogen-functionalized heterogeneous TiO_2_ homojunctions as the sensor layer (N-MXene) with urea solvent heat treatment at different reaction times to detect trace NH_3_ gas at room temperature (20 °C). The results show that the sensor (N-MXene-18) treated for 18 h displayed stronger response, fuller recovery, and faster response/recovery speed in wet environments than in dry environments, indicating the significant effect of humidity on the sensor performance (Figure 13a). As for the accelerated reaction kinetics, on the one hand, conducting paths constructed by water dissociation were beneficial for carrier transfer. On the other hand, NH_3_ molecules could reversibly dissolve into and out of the water and promote a rapid adsorption/desorption balance. At the same time, the pre-adsorbed water probably occupied partial high-energy adsorption sites that were difficult to restore due to a strong interaction with gas molecules, thus facilitating a full recovery. Similarly, Wang et al. [176] also found that the response and recovery speeds of the prepared MoS_2_-PEO sensor were accelerated with increasing RH.

#### 3.5.2. Specific Carrier Gas Assistance

There are also other intriguing ways to enhance the sensor response/recovery. For example, Ko et al. [177] synthesized a large and uniform WSe_2_ layer nanostructure. The carrier gas was converted from pure N_2_ to a gas mixture of NH_3_ and N_2_ (Figure 13b). This modification dramatically reduced the recovery time from 85 min to 43 s at room temperature after exposure to 500 ppm NO_2_ (Figure 13c). The enhanced response/recovery was related to NO_2_ and NH_3_ response on the WSe_2_ surface, similar to the mechanism of selective catalytic reduction (SCR) (Figure 13d) [178,179]. Namely, NH_3_ was adsorbed on the surface of WSe_2_ during the stabilization process within the carrier gas of the N_2_/NH_3_ mixture. Upon the initial state of NO_2_ introduction, pre-adsorbed NH_3_ reacted with NO_2_ to produce N_2_ and water. Due to the weak adsorption energy, the remaining NH_3_ was easily desorbed, and NO_2_ was then adsorbed to undergo the gas-solid reactions. During the recovery process, NH_3_ gas in the carrier gas reacted with the adsorbed NO_2_ to form N_2_ and water. After this reaction, the residual NH_3_ molecules on the surface of WSe_2_ waited for the subsequent gas-solid reactions in the next cycle.

#### 3.5.3. Gate Voltage-Assistant Technology

From the view of convenient sensor integration, gate voltage-modulation is an ideal strategy to assist the recovery speeds of gas sensors, especially for field effect transistor (FET)-type sensors. As the channel materials in this sensor structure always present a detectable conductivity variation during the adsorption/desorption of target gas molecules, we categorize FET sensors as one type of conductometric gas sensor. Graphene and carbon nanotube-based FET gas sensors have been favored by many researchers due to their advantages of short recovery time, low manufacturing cost, and scalability [180,181]. For example, Novak et al. [182] achieved a rapid recovery by applying a positive Si gate voltage for 200 s to single-walled carbon nanotube (SWCNT) networks based on dimethyl phosphonate (DMMP) transistors. Chang et al. used the FET sensor with carbon nanotubes as the channel material to detect NO_2_ and NH_3_ and realized rapid recovery by using negative and positive gate voltage, respectively [183,184]. Jaiswal et al. [185] doped multilayered graphene (MLG) with iodine and significantly reduced the recovery time by ~88% after applying a negative gate voltage of −15 V.

Wu’s team [186] found that a positive biased voltage (Vpb) could reduce the recovery time of SnO*_x_* sensors against oxidizing gases (NO_2_). To verify that the opposite Vpb (i.e., negative Vpb) imposed the same effect on the detection of reducing gas (H_2_S), they deposited a 200nm-thick layer of SnO_x_ as the sensing material on the interlocking horizontal control gate (CG) and a floating gate (FG), applying a negative pre-bias condition to CG prior to the sensor operation [187]. The research results showed that when the Vpb value of the sensor at 180 °C was 1 V, a long recovery time was obtained toward 2 ppm H_2_S. As Vpb drops from 0V to −3 V, the recovery time was gradually dropped to 960 s. With a pre-bias of −3 V, the sensor’s recovery time toward 2 ppm H_2_S was dramatically reduced by 74%. Due to the Ohmic contact between N-type SnO*_x_* and CG metals being produced, applying a negative Vpb to SnO*_x_* caused the band of SnO*_x_* to bend downward at the interface as compared to the natural state (Vpb = 0). As more electrons accumulated at the interface, more oxygen molecules were ionized per unit of time, and this resulted in a faster recovery.

## 4. Conclusions and Outlook

In conclusion, this paper reviews the existing factors that affect the response-recovery speeds of MOS-based CGS and proposes some relevant improvement strategies. The selection of gas-sensitive material, including its grain size or morphology, strongly correlates with the response/recovery speeds of relevant gas sensors. Various improvement methods, such as external heat and light excitation, the synthesis of porous and hierarchical structures, element doping, and composite engineering, are discussed in detailed. Some intriguing methods, including humidity introduction, pre-adsorbing other gas molecules on the sensing material, and gate-voltage modulation, are also mentioned.

From the view of the binding strength between sensing materials and gas molecules, strong adsorption means a large response, while weak desorption and difficult regeneration. This consequently raises the issue of a trade-off between sensitivity and recovery ratio. At present, increasing the operating temperature of the gas sensors seems to be the most effective way to accelerate the sensor response and recovery, but at the cost of the response intensity in some cases. The response of MOS-based gas sensors as a function of operating temperature is generally volcanic. In addition, heat energy usually comes from an external heater and thus complicates the design of the measurement device. Moreover, there are some application scenario limits with these heating-activated gas sensors. When detecting some flammable gases (such as H_2_, CO, methane, etc.) with a concentration higher than its explosion limit, safety risks are extremely induced by igniting the combustible gas and triggering explosion accidents. More important but easily ignorant is that prolonged heat aging readily alters the crystalline structure and deteriorates the sensor performance, such as repeatability and long-term stability.

The essential of light irradiation is to enhance the electron exchange between gas molecules and sensitive materials by using the photosensitive properties of gas-sensitive materials. As a result, the range of available materials is severely limited. In addition, long-term high-energy irradiation, especially UV light, will bring about a serious photo-corrosion effect. Then the intrinsic physicochemical properties of materials will be changed, which will be especially unfavorable to the long-term testing and utilization of related sensors. Future research could focus on visible light-assisted gas sensing, and additional efforts are required to design the electronic structure of the dual gas/light-sensitive materials to boost energy utilization effectively.

Although the modulation effect of the thickness, grain size, and microstructure are remarkable, the increasing synthesis difficulties cannot be ignored. Moreover, it is often laborious to control the uniformity and reproducibility in the synthesis/regulation of specific nanostructures, which seriously affects the performance consistency between different batches of sensors and delays the summary and design of reliable improvement schemes.

For element doping, additional attention should be paid to the utilization efficiency of external elements. Appropriate element loading should be taken into account, as a low loading will limit the performance gains, and too much can make it difficult to avoid element agglomeration. In recent years, the relevant literature has reported single-atom catalysts (Pd, Pt, Au, etc.) for gas sensing [188,189], which have broad application potential in speeding up the response and recovery and need more exploration.

The key to constructing composite materials is the formation of heterogeneous interfaces. The effective control of carrier transport within the interfaces is critical to improving the response/recovery speeds. More design and analysis are required because the mechanisms involved are still unclear. With the help of spectroscopic methods such as photoluminescence (PL) emission spectra, the generation and attenuation rates of photocurrent with different heterojunction structures could be explored, and the carrier transfer characteristics at the interfaces can also be roughly comprehended [190,191].

A lot of research groups have also tried to use a combination of these methods (such as doping, composite engineering, nanostructure modulation, etc.) to reduce the working temperature of gas-sensitive materials while achieving a rapid recovery, but the difficulties remain. In addition to binary composites of metal oxides and other metals or semiconductors, ternary composites are increasingly exploited. However, clarifying the underlying sensing mechanisms of these nanocomposites was very complicated. Therefore, more endeavors should be undertaken in this field to pave the way for future composite design so as to achieve quicker response and recovery.

The preparation of ordered macroporous oxide nanostructures is a promising direction for future research, including two-dimensional (2D) and three-dimensional (3D) ordered macro-mesoporous nanostructures and aperture-controllable 3D interconnected macro mesoporous nanostructures [192]. Studies have shown that ordered macroporous nanostructures can significantly promote gas diffusion, thus improving sensing response and dynamics, which is conducive to accelerating gas sensing. The combination of macro–mesoporous structures can not only provide highly connected diffusion channels but also generate surface accessibility, facilitating fast sensing kinetics and high gas response [84,193].

Gas sensors based on suspended materials also provide a way of setting the future direction for accelerating gas-sensitive reactions [194]. Gas molecules could diffuse and penetrate the sensing layer from the top and the bottom sides, avoiding interfacial scattering effects and expediting the adsorption/desorption balance. Sensors with suspended materials can improve the performance of gas sensors to theoretical limits and lead to faster response and recovery than those in the supported ones [195].

In the process of concluding the latest research advances of accelerating the reaction speeds of MOS-based gas sensors, we realized that the efforts in this field still need more enrichment. In addition, more specialized theories, such as molecular dynamics and carrier transport, should be combined with the reaction speeds of CGS so as to shed light on the underlying mechanisms. Therefore, this review is expected to enlighten readers to explore further novel gas sensors especially featuring swift response and recovery.

## Figures and Tables

**Table 1:** Typical sensing characteristics of representative sensors to 5 ppm NO_2_ at 30 °C in dry air under UV light irradiation (365 nm) of 7 mW cm^−2^.

Photoactive Material	Resistance in Air (Ω)	Crystal Structure	Specific Surface Area (m^2^ g^−1^)	Response (Rg/Ra)	T_res_/T_rec_ (min)
SnO_2_	1.2 × 10^4^	Tetragonal	21.2	360	4.6/1.6
In_2_O_3_	2 × 10^2^	Cubic	23.7	22	3.9/1.0
WO_3_	3.1 × 10^5^	Monoclinic	12.0	7.2	12.2/7.0

**Table 2:** List of various main efforts applied to accelerate response/recovery of gas sensors.

Main Accelerating Method	Material	Gas/Conc. (ppm)	T (°C)/W(nm) /I(mW⋅cm^−2^)	Response (S)	Tres/Trec	LOD (ppm)	Ref.
External excitation	TiO_2_ nanotube array films	H_2_S/1	300/-/-	4.5	9 s/6 s	1	[3]
	Au decorated hierarchical ZnO	Acetone/100	340/-/-	112.3	4 s/6 s	-	[139]
	Ag functioned WO_3_ nanosheets	HCHO/100	300/-/-	20.83	5 s/5 s	-	[50]
	Porous In_2_O_3_ microstructures	Cl_2_/50	300/-/-	~905	2 s/4 s	-	[140]
	LaCoO_3_ modified ZnO	Ethanol/100	320/-/-	55	2.8 s/9.7 s	0.5	[141]
	Dye-sensitized POM/TiO_2_ films	NO_2_/1	RT/480/-	231	48 s/66 s	0.05	[51]
	La-coated ZnO nanorods	H_2_/100	RT/365/-	63.80%	15 s/9 s	-	[142]
	WS_2_ nanosheets/SnO_2_ QDs 2D/0D heterostructures	NO_2_/5	RT/365/0.37	340%	10 s/9 s	0.5	[54]
	rGO decorated TiO_2_ nanoplates	NO_2_/100	RT/365/5.34	35.60%	~59 s/33 s	0.11	[55]
Nanostructure design	porous α-Fe_2_O_3_ nanotubes	Acetone/100	240/-/-	11	9 s/3 s	-	[143]
	In_2_O_3_ hierarchical architectures	HCHO/100	260/-/-	8.6	1 s/8 s	1	[86]
	α-Fe_2_O_3_ Nano-Ellipsoids	H_2_S/50	260/-/-	8	0.8 s/2.2 s	0.1	[144]
	Mesoporous In_2_O_3_	H_2_/500	260/-/-	18	1.7 s/1.5 s	0.1	[145]
	Vertical SnO*_x_* nanopillars	NH_3_/2.2	RT/-/-	-	2.7 s/24.2 s	1	[146]
	Hierarchical Co_3_O_4_ micro rods	Methanol/100	220/-/-	14	0.8 s/7.2 s	-	[38]
	Hierarchical porous SnO_2_	Ethanol/20	260/-/-	~64	10 s/5 s	-	[147]
Element doping	W-doped SnO_2_ nanoparticles	H_2_S/10	260/-/-	3.6	17 s/7 s	0.1	[77]
	La-doped SnO_2_ nanoparticles	Methanol/75	220/-/-	29.5	12 s/7 s	-	[98]
	Gd-doped Co_3_O_4_ nanoparticles	O_2_/40,000	240/-/-	921%	23 s/22 s	-	[14]
	Pd_7.18%_W_18_O_49_ nanowires	Acetone/50	175/-/-	~150	5 s/10 s	0.3	[93]
	Ag-doped ZnO thin films	NH_3_/100	RT/-/-	8260%	27 s/7 s	-	[99]
	Pr-doped SnO_2_ hollow tubes	Ethanol/100	200/-/-	35.6	12 s/8 s	2	[148]
	Coral-like Sm-doped PrFeO_3_	Acetone/50	270/-/-	44.94	15 s/16 s	-	[149]
	Y-doped SnO_2_ hierarchical nanoflowers	HCHO/50	180/-/-	18	8 s/10 s (25 ppm)	1	[150]
	Co-doped sponge-like In_2_O_3_	Acetone/100	240/-/-	32.8	~1.1 s/37.5 s	5	[49]
Composite engineering	α-Fe_2_O_3_/SnO_2_ nanowires arrays	Toluene/100	90/-/-	49.70%	20 s/15 s	-	[151]
	ZnO/V_2_O_5_ thin films	Toluene/400	27/-/-	~2.3	23 s/28 s	-	[152]
	SnO_2_-BiVO_4_ heterojunction	NO_2_/0.1	RT/-/-	0.91%	13 s/9 s	0.1	[105]
	α-Fe_2_O_3_ loaded rGO nanosheets	CO/10	RT/-/-	48.14%	21 s/8 s	-	[153]
	Fe_2_O_3_-loaded NiO nanosheets	Methanol/100	255/-/-	107.9	0.1 s/11.4 s	-	[154]
	Porous CuO/ZnO tubule	H_2_S/0.05	170/-/-	~1.6	35 s/29 s	0.01	[155]
	Hierarchical SnO/SnO_2_ 3D nanoflowers	HCHO/50	120/-/-	80.9	7 s/27 s	0.008	[156]
	Highly porous SnO_2_-CuO nanotubes	H_2_S/5	200/-/-	1395	5.27 s/40 s	-	[37]
	MoS_2_ nanosheets/multilayer WS_2_ heterojunction	NO_2_/50	RT/-/-	27%	1.6 s/27.7 s	0.01	[157]
	SnO_2_ nanorod decorated WSe_2_ nanosheets heterojunctions	NH_3_/5	RT/-/-	87.07%	24 s/40 s	0.1	[111]
	WO_3_ nanoparticles/multi-layer graphite nanocomposite	2-CEES/5.7	260/-/-	63%	8 s/34 s	0.1	[4]
	Planar rose-like ZnO/HGaN heterojunction	H_2_/50	150/-/-	15.82	47 s/6 s	5	[158]
	SnO_2_ nanoflowers/rGO composites	NO_2_/0.00001	RT/-/-	10.50%	59 s/9 s	0.00001	[159]
	Nanowire bundle-like WO_3_-W_18_O_49_	NH_3_/500	250/-/-	23%	13 s/49 s	0.46	[160]
	Multilayer MXene decorated SnO_2_ microspheres	Ethanol/10	230/-/-	5	14 s/26 s	0.5	[161]
	Macroporous flower-like structured CdS/CdIn_2_S_4_ het erojunctions	Triethylamine/10	161/-/-	32.5	3 s/256 s	0.5	[162]
	Porous CaFe_2_O_4_/ZnFe_2_O_4_ heterojunction composites	Isoprene/30	200/-/-	19.5	72 s/35 s	-	[163]
	graphene QD-modified SnO_2_ cubes	NO_2_/1	130/-/-	417	59 s/33 s	0.2	[164]
	3D α-Fe_2_O_3_ nanorods @GO nanosheets	Acetone/50	220/-/-	19.14	7 s/8 s	--	[130]
	Bilayered TiO_2_/ITO films	H_2_/200	RT/-/-	~1.1	-/4 s	-	[165]
	Au-decorated SnO_2_ nanoparticles	n-buthanol/200	240/-/-	251	3 s/11 s	1	[166]
	Au-loaded multilayered SnO_2_ nanosheets	CO/50	220/-/-	36.5	1 s/4.1 s	1	[167]
	In/Pd co-doped SnO_2_ microspheres	HCHO/100	160/-/-	24.6	3 s/6 s	-	[168]
	Carbon nanotubes decorated NiO/SnO_2_ composite nanofibers	Acetone/50	160/-/-	25.25	8.2 s/10.5 s	-	[42]
	SnO_2_:CuO nanoparticles within macroporous silicon layer	NH_3_/150	RT/-/-	57%	4 s/55 s	-	[169]
	Core-shell Au@NiO/SnO_2_ microspheres	Acetone/100	300/-/-	49.7	4 s/5 s	0.5	[170]
	Pd coated SiO_2_/Si nanostructures	H_2_/10,000	RT/-/-	88%	1.4 s/14 s	-	[171]
	PdO decorated ZnO/ZnCo_2_O_4_ heterostructured microsphere	HCHO/100	139/-/-	26.9	9 s/14 s	0.2	[172]
	La_2_O_3_-modified SnO_2_-Sn_3_O_4_	HCHO/100	220/-/-	117.27	3 s/3 s	0.08	[173]

Conc.: Concentration; T (°C)/W(nm)/I(mW·cm^−2^): Operating temperature (°C)/Light wavelength (nm)/Light intensity(mW·cm^−2^); S: Ra/Rg or Rg/Ra or |(Rg-Ra)|/Ra; LOD: Limit of detection; TEA: Triethylamine; SM: Sulfur mustard; ITO: Indium tin oxide; POM: Polyoxometalate; RT: Room temperature; 2-CEES: 2-chloroethyl ethyl sulfide; QD: Quantum dots.

## Data Availability

Not applicable.
